# *Burkholderia* Aortic Aneurysm: A Case Report and Review of the Literature

**DOI:** 10.1155/2017/6206395

**Published:** 2017-11-07

**Authors:** Sreelakshmi Panginikkod, Aishwarya Ramachandran, Pratyusha Bollimunta, Roshanak Habibi, Roshan Kumar Arjal, Venu Gopalakrishnan

**Affiliations:** ^1^Division of Internal Medicine, Presence Saint Francis Hospital, 355 Ridge Ave., Evanston, IL 60602, USA; ^2^Division of Radiology, Presence Saint Francis Hospital, 355 Ridge Ave., Evanston, IL 60602, USA

## Abstract

Melioidosis is a frequently fatal infection caused by the Gram-negative bacillus *Burkholderia pseudomallei* endemic to Southeast Asia and Northern Australia. It is a rare imported pathogen in the United States and is a potential bioterror agent. We report the case of an 82-year-old previously healthy man who presented with 2 weeks of fever and epigastric pain after he returned from the Philippines. A diagnosis of nondissecting mycotic aneurysm in the descending thoracic aorta was made with the help of CT angiogram and positive blood cultures. The patient completely recovered with a 6-month antibiotic therapy followed by surgical repair of the aneurysm. Given the slight increase in the number of melioidosis cases reported by CDC since 2008, melioidosis might be considered an emerging infectious disease in the United States. The purpose of this report is to raise awareness of the disease among clinicians as well as travelers.

## 1. Introduction

*Burkholderia pseudomallei* is a widely distributed environmental saprophyte causing serious infections in endemic regions of Southeast Asia and Northern Australia but is rarely reported in the United States [1]. They are predominantly transmitted through direct contact with an environmental source (wet soil or contaminated water) by ingestion, percutaneous inoculation, or inhalation of the bacterium. Common manifestations of melioidosis include pneumonia, skin abscesses, ulcers, osteomyelitis, and septic arthritis. However, mycotic aneurysm is a rare presentation found only in 1%–2% of cases and is related to high rates of morbidity, mortality, and relapse [2]. Due to its severe impact on human health and potential to transmit through inhalation, *B. pseudomallei* is considered a biological threat as well as a potential bioterror agent. We herein report a case of *Burkholderia*-associated thoracic aortic aneurysm from a community hospital in the United States.

## 2. Case Report

An 82-year-old man presented to our hospital with 2 weeks of fever, anorexia, drenching sweats, and epigastric pain radiating to the back. He denied any nausea, vomiting, or change in bowel habits. He visited the Philippines 4 months prior to presentation and did not have any sick contacts. Medical history was relevant for hypertension, hyperlipidemia, and osteoarthritis. He is a nonsmoker, nonalcoholic, and did not have similar episodes in the past.

**Figure 1:**
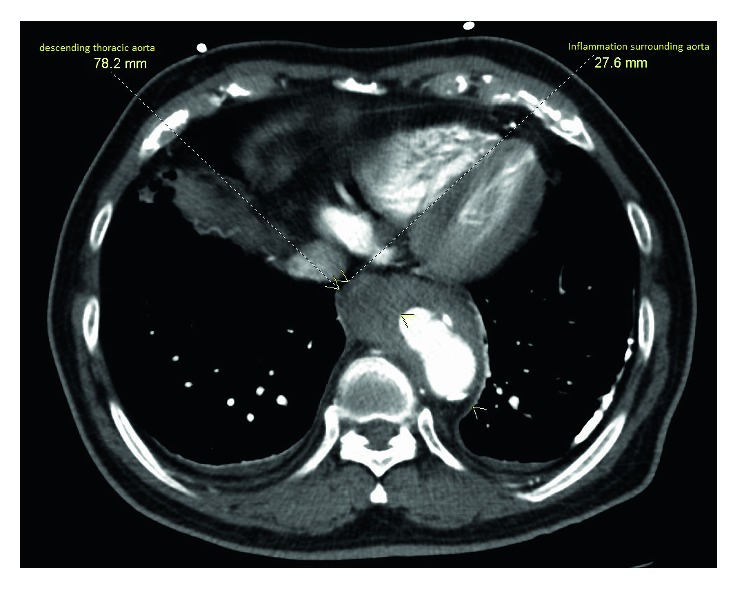


On admission, he was febrile to 101°F with a normal blood pressure (154/76 mm Hg), heart rate (86 bpm), respiratory rate (16 breaths per minute), and oxygen saturation (>95% on room air). There were no peripheral stigmata of infective endocarditis. Abdomen examination was significant for epigastric tenderness with no palpable mass or pulsations and normal bowel sounds. Examination of other systems were unremarkable. Routine labs showed a hemoglobin level of 14.6 gm/dl, white blood cell (WBC) count of 5.2 × 10^3^ per mm cube, normal liver enzymes (ALT 44 IU/L and AST 34  IU/L), and normal renal function (creatinine level 0.82 mg/dL). The lipid profile included a low-density lipoprotein cholesterol level of 121 mg/dl, triglyceride level of 112 mg/dl, and high-density lipoprotein cholesterol level of 48 mg/dl. Urinalysis was normal. C-reactive protein level was elevated (44 mg/L). Quantiferon gold, HIV, and hepatitis tests were negative. Autoimmune panel was also negative. Chest radiography showed tortuous aorta with multiple aortic calcifications. CT abdomen showed saccular outpouchings from the descending thoracic aorta just above the diaphragm concerning for penetrating atherosclerotic ulcers with periaortitis (Figure 1). In order to further differentiate the esophagus from aneurysm, he underwent a CT angiogram of the chest with a small amount of oral contrast which demonstrated distal thoracic aorta aneurysm with no evidence of leak or hematoma. In the absence of a clear etiology for fever, he was started on vancomycin and ceftriaxone. On day 3, blood cultures drawn on the day of admission prior to antimicrobial therapy grew *Burkholderia pseudomallei*. An extensive septic screen, including urine culture, sputum culture, and transthoracic and transesophageal echocardiograms revealed no abnormalities.

The patient refused surgical management initially and was started on intravenous ceftazidime based on sensitivity results (Table 1). His fevers remitted, multiple repeat blood cultures remained sterile, and CRP returned to normal within 1 week. He was discharged on an additional 6-week course of ceftazidime 2 g BID through peripherally inserted central catheter followed by 3 months of oral Bactrim.

**Figure 2:**
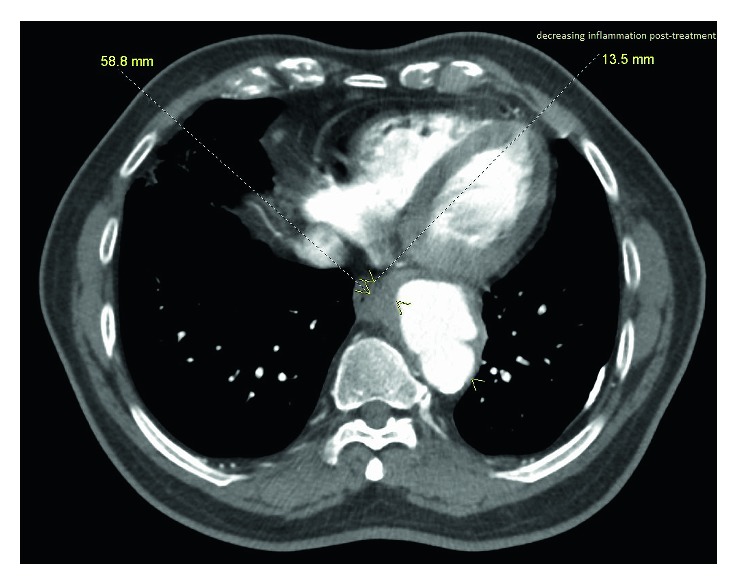


Surveillance CT angiogram at 5 months redemonstrated mycotic aneurysm of the distal descending thoracic aorta with decrease in mural thickening of the sac and improved inflammation of the adjacent posterior mediastinal fat (Figure 2). CT-PET after completion of antibiotics showed minimal signal in the aneurysm, consistent with microbiological suppression. Even though the bacteremia cleared, the aneurysm persisted, and hence, surgery was offered again as the only curative option. The patient agreed, and elective endovascular repair of aortic aneurysm was subsequently conducted without any complication.

## 3. Discussion

Melioidosis is a severe infectious disease caused by *Burkholderia pseudomallei* and was first described in Burma by Captain Alfred Whitmore. Since then, it has been known as a causative agent of community-acquired bacteremia in endemic countries such as Taiwan, Singapore, and Malaysia [1]. Mycotic aneurysm, a localized and irreversible dilatation of an artery caused by infection, is a rare presentation of melioidosis. The first description of mycotic aneurysm caused by *B. pseudomallei* was reported in 1998 in a 70-year-old man with hypertension [3]. Regional conditions and endemic diseases determine the etiology of mycotic aneurysms. In temperate areas, the *Burkholderia* infection is extremely rare and is almost always imported by travelers or immigrants. It has a potential latency of infection and may present itself years after exposure. Our patient returned from the Philippines recently and might have been exposed to this organism during his travel.

In a 20-year prospective study of 540 cases of melioidosis in Northern Australia reported by Currie et al., only 2 patients had mycotic pseudoaneurysm [4]. In that study, about 80% of patients had risk factors including diabetes mellitus (39%), alcohol abuse (39%), chronic lung disease (26%), chronic renal disease (12%), rheumatic heart disease and/or congestive heart failure (7%), malignancies (6%), and immunosuppression (6%) [4]. In another study of infected aortic aneurysms in an endemic area for melioidosis, the most common comorbidities identified were hypertension and renal disease with some degree of atherosclerosis also playing a role [5]. In accordance with these studies, our patient was hypertensive and also had atherosclerotic changes in aortogram.

The pathogenesis of *B. pseudomallei* infection in mycotic aneurysm is marked neutrophilic inflammation and microabscess formation. This process and its progression are facilitated by the resistance of this intracellular bacterium to polymorphonuclear leucocytes [6].

Establishing the diagnosis of mycotic aneurysm due to *B. pseudomallei* can be challenging due to its nonspecific presentation and rare incidence. It should be considered in febrile patients with risk factors for atherosclerosis who have ever been to endemic areas and have (i) abdominal, back, or thigh pain; (ii) vertebral, paravertebral, or retroperitoneal collections; (iii) relapse of melioidosis; or (iv) persistently positive *B. pseudomallei* blood cultures [6]. Computed tomography (CT) angiogram is the diagnostic modality of choice. In our patient, the aortic aneurysm was detected on the day of presentation itself with the help of CT angiogram. But, the mycotic etiology was unraveled only on the third day when the blood cultures came positive. Until then, extensive infectious and autoimmune workup was performed for his unexplained fever, and he was being treated with broad-spectrum antibiotics.

**Figure 3:**
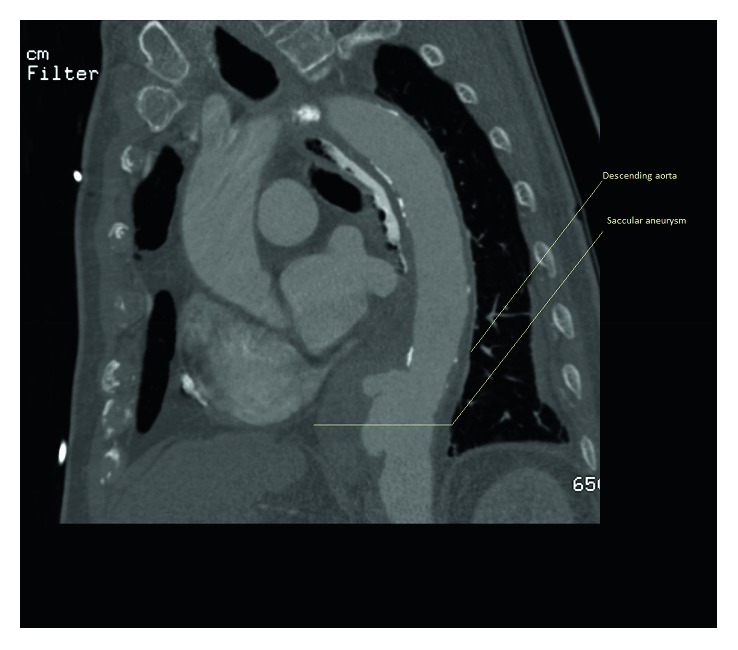


The CT appearances of mycotic aneurysms are categorized into four grades: grade 1: periarterial changes without destruction of the arterial wall; grade 2: presence of saccular outpouching; grade 3: extensive retroperitoneal infection; and grade 4: massive perianeurysmal hemorrhage [7]. Our patient had a grade 2 aneurysm as given in Figure 3.

*Burkholderia pseudomallei* is resistant to aminoglycosides due to an efflux system which prevents the accumulation of the antibiotic in the bacterium [8]. The currently recommended antibiotic regimen for mycotic aneurysm in melioidosis includes 4–6 weeks of high-dose intravenous ceftazidime or imipenem/meropenem followed by oral maintenance therapy usually with TMP-SMX as a backbone for 3–6 months [6, 9]. This was successful in clearing the bacteremia and reducing inflammation around the aneurysm prior to surgical repair in our patient.

Historically, the gold standard of treatment is wide surgical debridement and in-situ or extra-anatomical repair. Prior studies have mentioned high rates of post-op complications in *B. pseudomallei* mycotic aneurysm as compared to those caused by other pathogens, even with optimal surgical and medical management. Mortality rates of up to 40 percent are associated with open surgical repair [10–12]. This poor outcome may be a result of the plethora of medical comorbidities, magnitude of surgical insults, and presence of sepsis encountered in these patients.

In conclusion, we report a case of successful recovery of *Burkholderia* mycotic aneurysm with intense antibiotic therapy followed by surgical repair. The number of melioidosis cases reported in the United States have been increasing slightly each year since 2008. This might reflect an increase in travel to locations endemic for melioidosis or could represent unidentified foci of locally acquired *B. pseudomallei* infections in the United States [13]. Physicians should be aware of this increase in cases reported in the United States and consider possibility of melioidosis not only in patients hailing from endemic areas [14, 15] but also in patients returning from travel in those regions [14]. Early intervention is critical in reducing the mortality rate from this disease, which can be up to 90% in septic patients with delayed diagnosis and treatment.

## Figures and Tables

**Table 1:** The antibiotic sensitivity test.

Antimicrobial	MIC	Interpretation
Piperacillin + tazobactam	≤16	Resistant
Gentamicin	>8	Resistant
Amikacin	>32	Resistant
Tobramycin	>8	Resistant
Aztreonam	>16	Resistant
Ciprofloxacin	2	Intermediate
Ceftazidime	4	Sensitive
Imipenem/cilastatin	≤4	Sensitive
Meropenem	≤4	Sensitive
Cefepime	>16	Resistant
Trimeth-sulfa	≤2/38	Sensitive
